# Sediment Characteristics Determine the Flowering Effort of *Zostera noltei* Meadows Inhabiting a Human-Dominated Lagoon

**DOI:** 10.3390/plants10071387

**Published:** 2021-07-06

**Authors:** Laura Guerrero-Meseguer, Puri Veiga, Leandro Sampaio, Marcos Rubal

**Affiliations:** 1 Interdisciplinary Centre of Marine and Environmental Research (CIIMAR) of the University of Porto, Novo Edifício do Terminal de Cruzeiros do Porto de Leixões, Avenida General Norton de Matos, 4450-208 Matosinhos, Portugal; puri.sanchez@fc.up.pt (P.V.); leandro.sampaio@fc.up.pt (L.S.); marcos.garcia@fc.up.pt (M.R.); 2 Department of Biology, Faculty of Sciences, University of Porto, Rua do Campo Alegre s/n, 4169-007 Porto, Portugal

**Keywords:** seagrass, sexual reproduction, anthropogenic stressors, sediment, organic matter

## Abstract

Recent studies have shown increasing *Zostera noltei* meadows in areas modified by anthropogenic activities. However, it is not entirely clear whether this trend of expansion could be linked to a greater reproductive effort in the species. Anthropogenic stressors can induce the reproductive effort of seagrass meadows as a response to stress, but other variables, such as seagrass biometrics or environmental factors, can also influence their sexual reproduction. To increase the knowledge regarding this issue, we monitored the flowering effort, seagrass biometrics and abiotic parameters of three *Z. noltei* meadows in an area that has been highly modified by anthropogenic activities during the past decades. Results showed that silt and clay content in the sediment (strongly correlated with organic matter) and seagrass vertical shoot density explained 54% of the variability in the flowering effort of the meadows. This study suggests that stress-induced flowering of *Z. noltei* may occur under determinate environmental conditions, such as silty environments with organic enrichment.

## 1. Introduction

Seagrasses establish key ecosystems around the world, playing important ecological roles [1]. Among others, seagrass meadows preserve the coastal geomorphology, are responsible for seawater quality and clarity, and provide shelter, nursery and feeding areas for numerous marine organisms [2,3,4]. Although seagrasses cover only 0.1% of the world’s ocean floor, they significantly contribute to its primary production and carbon sequestration [5,6,7,8,9], but have been threatened by anthropogenic stressors over the past decades [10].

Seagrasses can inhabit intertidal and subtidal areas of estuaries and lagoons [10]. In recent decades, the influence of human activities such as aquaculture, dredging, wastewater or stormwater runoffs, shellfish harvesting and boat transit have strongly modified these ecosystems’ functioning [11,12]. The above-mentioned anthropogenic activities can cause, among others, mechanical impacts across the seagrass meadows and changes in their sediment composition [13,14,15]. Nevertheless, recent studies suggest that some seagrass species, especially of the genus *Zostera*, are currently adapting to human-modified environments [16,17,18,19,20,21,22].

*Zostera noltei* Hornemann is one of the seagrass species that best tolerates human-dominated environments. This seagrass shows characteristics of opportunistic species, displaying a high shoot turnover and forming seed banks in the sediments, which allow it to quickly recover after unfavorable periods or anthropogenic disturbances [1,23]. In addition, the species inhabits a wide range of salinities (7–35 psu) [24,25], can tolerate elevated seawater temperatures (above 37 °C) [26] and adapts to high light exposure conditions during low tide [27]. Thus, *Z. noltei* is suited to intertidal areas of estuaries and lagoons, such as Ria de Aveiro, which are also environments dominated by several human activities [16,20]. Numerous studies have shown that there is a positive trend in this seagrass expansion amidst environments dominated by human activities during recent decades (i.e., shellfish harvesting and dredging) [16,20]. Moreover, the flowering effort of *Z. noltei* can increase under the influence of certain anthropogenic perturbations such as mechanical, sedimentary and hydrodynamic impacts [28,29,30]. However, the reproductive capacity of *Z. noltei* in human-dominated areas where the species is expanding has not yet been evaluated. Although seagrass expansion can be maintained only by asexual reproduction of its own rhizomes, sexual reproduction sustains the long-term survival of the species when vegetative growth is limited [31,32,33]. In addition, sexual reproduction on seagrasses provides genetic diversity, which is essential for clonal organisms since it allows them to improve their survival when facing upcoming stressors [34,35]. Thus, understanding the sexual reproduction of *Z. noltei* in human-dominated environments could provide some insight about the expansion drift of this species.

Modifications in abiotic parameters (i.e., temperature, salinity and light intensity) can influence the timing and intensity of the flowering in *Zostera* species, leading to high spatial variability in their reproductive effort [36,37,38]. At high latitudes, the flowering of *Z. noltei* starts in the hottest spring months and ends when the temperatures start to drop, by the end of autumn [36,39]. Although the mechanisms that control sexual reproduction in this seagrass are not yet fully understood, it is known that its flowering effort can vary under different sediment types, colonization stages and vegetative growth capacity [28,29,40,41]. Therefore, evaluating the flowering of *Z. noltei* in an environment with great spatial variability and subject to multiple and simultaneous anthropogenic stressors, such as Ria de Aveiro, can help us forecast the future of impacted meadows and understand which factors could influence the stress-induced flowering response of this seagrass.

The aim of this study was to determine if seagrass biometrics and environmental factors could influence the flowering of human-dominated *Z. noltei* meadows. To test this, the flowering effort, seagrass biometrics and abiotic parameters of three *Z. noltei* meadows inhabiting an area subjected to several anthropogenic activities (dredging, oyster culture, stormwater runoffs and bait digging) were measured during the period of sexual reproduction. The relationship between the flowering effort of the species and biometric and abiotic parameters was also analyzed.

## 2. Material and Methods

### 2.1. Study Area

**Figure 1:**
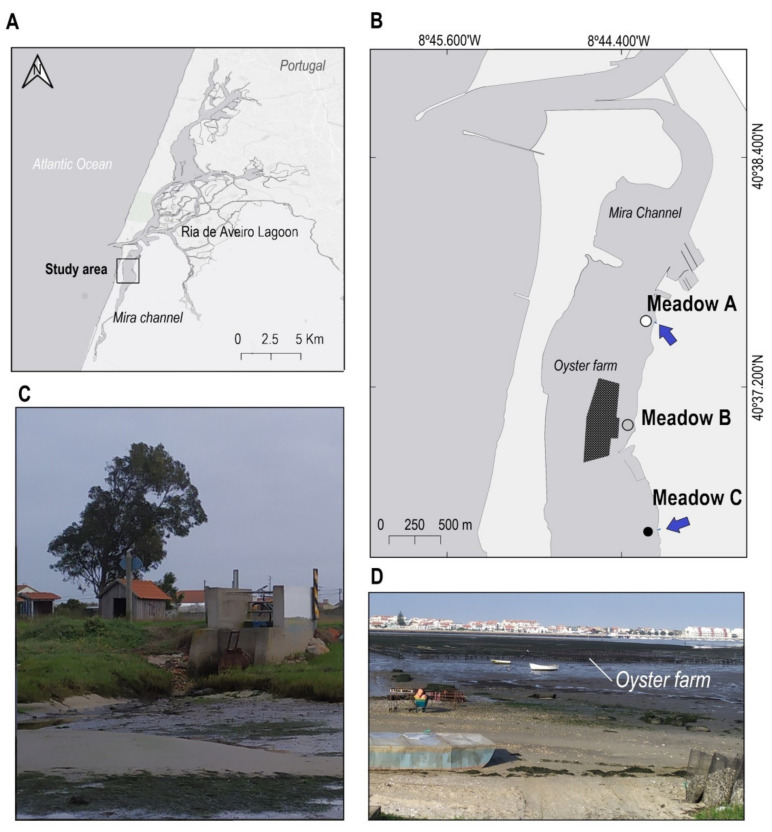
Study area (Ria de Aveiro, Center of Portugal) (**A**) and location of the three *Z. noltei* meadows in the Mira Channel (**B**). The dark area in (**B**) indicates the location of the oyster farming, while the blue arrows indicate the position of the storm drain outlets. The white, grey and black spots of (**B**) show the location of each *Z. noltei* meadow. (**C**) shows the storm drain outlet and runoff of the Meadow C. (**D**) displays the oyster farm trestles and farming activities in Meadow B.

The present study was done in the Mira channel of the Ria de Aveiro lagoon (Aveiro, Portugal; Figure 1A). This channel is an elongated and shallow arm, 25 km long, that runs south-southwest, parallel to the coastline (Figure 1A). During floods, only about 20% of the tidal prism is diverted to this channel, while a continuous freshwater supply is received in the upper part through a small system of lagoons and streams. This creates a salinity gradient during high tides, with very low salinities in the most internal areas (0−5 psu), high values at the mouth (25−36 psu) and highly variable salinity ranges in the middle section of the channel [42], where the study was conducted.

The Ria de Aveiro lagoon has been highly modified by human activities over the past decades, resulting in a spatial mosaic of different environmental conditions, especially regarding sediment composition. This lagoon is frequently subjected to dredging to prevent siltation and to maintain hydrodynamics of its channels, widely used for navigation and recreational purposes [43]. Dredging activities have increased the penetration of the tidal wave, enhancing turbidity and causing resuspension of coarse sandy sediments from the deepest areas of the lagoon and their deposition to the tidal flats nearby [44]. Other common anthropogenic pressures in Ria de Aveiro are fishing and related activities such as bait digging, shellfish harvesting or the use of motor boating, which have a high potential of disturbing the sediment [14]. The erosion of the sediments promoted by these anthropogenic activities is causing the loss of finer sediments, nutrients and organic matter content in the *Z. noltei* meadows of the lagoon, producing negative impacts in their development [45].

Apart from the above-mentioned anthropogenic activities, oyster aquaculture is also intensively developed in the Mira channel, and several storm drain outlets are discharging into this system (Figure 1B). Thus, three monospecific *Z. noltei* meadows were selected in the channel, encompassing an area where most of these anthropogenic activities occur (Figure 1B). Two of the meadows (Meadow A and Meadow C) were adjacent to the storm drain outlets (Figure 1C), and another (Meadow B) was close to an oyster farm (Figure 1D). The selected *Z. noltei* meadows were all intertidal and had similar depth, size area (between 4 and 6 ha) and patchiness [16]. Meadow A was the closest to the sea, followed by Meadow B and Meadow C (Figure 1A). The coverage of *Z. noltei* was higher in Meadow B (90.12 ± 6.037%) than in Meadows A and C, which showed similar values (68.37 ± 4.044% and 70.00 ± 10.32%, respectively).

### 2.2. Monitoring of Flowering Effort, Seagrass Biometrics and Abiotic Parameters

Monitoring was always carried out during low-tide periods from July to November 2019. The chosen interval coincides with the beginning and end of the flowering period of the three *Z. noltei* meadows. Four dates were chosen randomly during the flowering period (July, August, October and November) to determine temporal variability in the flowering effort, seagrass biometrics and abiotic parameters among meadows.

Flowering effort was determined by counting the sexual spathes in a 20 × 20 cm quadrat (n = 3) on each meadow and date. Seagrass biometrics (vertical shoot density, vertical shoot height, leaf area and maximum root length) were measured within the same quadrat used to measure the flowering effort. The vertical shoot density was determined by counting, in situ, the number of vertical shoots in three sub-quadrates of 10 × 10 cm. Subsequently, six *Z. noltei* ramets (vertical shoots containing roots and leaves) were randomly picked up within each 20 × 20 cm quadrat to measure the rest of the seagrass biometrics. Vertical shoot height was determined by measuring the distance from the bottom of the shoot to the base of the leaves. The leaves on each vertical shoot were counted and measured (width and length) to calculate the leaf area. In this way, leaf area was calculated by multiplying the leaf width by the sum of the leaf length per vertical shoot and dividing it by the total number of leaves per shoot. Maximum root length corresponded to the measurement of the largest root found at the base of each ramet.

Regarding abiotic parameters, seawater salinity and temperature, redox potential, sediment grain size and organic matter content were assessed in each meadow and date (n = 2). Seawater salinity and temperature were recorded using a portable meter (HQ40, Hach, Germany). Redox potential was determined in the sediments with a portable analog meter (HI 8314, Hanna, Smithfield, RI, USA). To determine sediment grain size and organic matter content, two sediment corers (3.7 cm diameter) were randomly collected within each meadow. Sediment grain size was determined by drying the sediment at 60 °C for 24 h and sieving it through different mesh sizes. Then, the sediment was classified following the Wentworth scale: fine gravel (2–4 mm), very coarse sand (1–2 mm), coarse sand (0.5–1 mm), medium sand (0.25–0.5 mm), fine sand (0.125–0.25 mm), very fine sand (0.063–0.125 mm), and silt/clay (<0.063 mm) [46]. Organic matter content was measured using 1 g of the finest fraction of the sediment resulting from this sieving (i.e., <0.5 mm), muffled at 450 °C for approximately 4 h, and estimated as the difference in weight before and after combustion.

### 2.3. Data Analyses

Temporal variability of the flowering effort, seagrass biometrics, and abiotic parameters among meadows was analyzed by using two-way ANOVA with *meadow* as fixed factor and *time* as random factor (July, August, October and November). Tukey’s multiple comparison tests were used to examine pairwise differences. Data were tested for normality and for homogeneity of variance using the Shapiro test and the Bartlett’s test, respectively. Whenever necessary, data were transformed to comply with the assumptions of ANOVA. The statistical α was adjusted to *p* < 0.01 for variables, which could not be transformed to meet parametric requirements [47].

To explore the relationship between flowering effort, seagrass biometrics and abiotic parameters, non-parametric multivariate multiple regression analyses [48] were used. Biometrics and abiotic parameters data were subjected to a stepwise forward selection procedure to develop a model for the flowering effort data by testing each seagrass biometric and abiotic variables. Analyses were based on Euclidean similarity matrices. P-values were done using 9999 permutations of residuals under the reduced model [49]. All non-parametric multivariate multiple regressions were done using the computer program DISTLM [50]. Draftsman plots were done beforehand to check for skewness in the biometric and abiotic variables. Silt/Clay and organic matter showed a strong correlation (r > 0.9); hence, organic matter was removed from the analyses and silt/clay was maintained, to avoid redundancy. Therefore, results obtained for silt/clay could be exchangeable with the organic matter content [50]. Finally, constrained ordination, a distance-based redundancy analysis (dbRDA) [51], was done to explicitly investigate the relationship between variables and flowering effort.

## 3. Results

### 3.1. Abiotic Parameters

**Figure 2:**
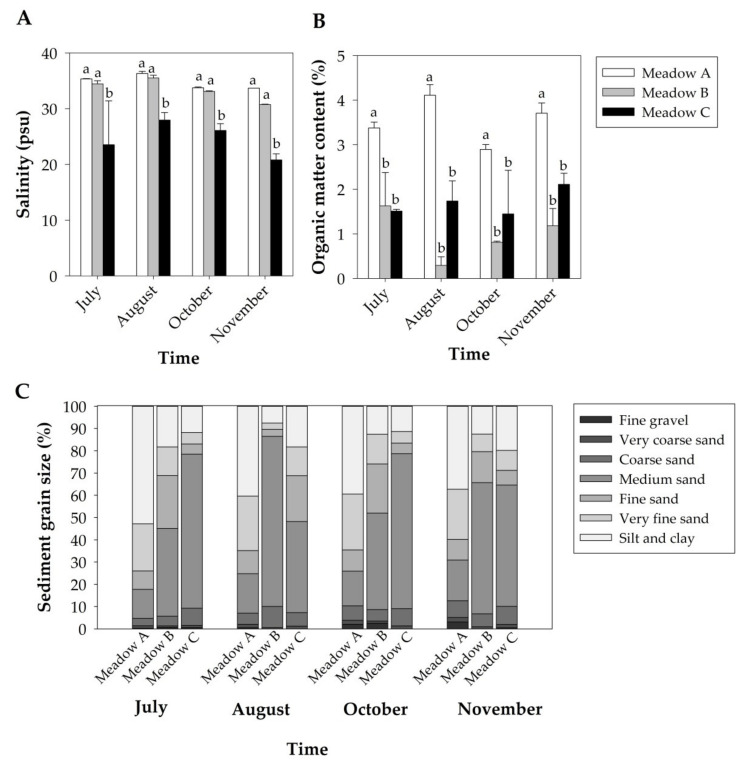
Abiotic parameters (Mean + SE, n = 2) of the studied *Z. noltei* meadows along the study period: seawater salinity (**A**), organic matter content of the sediment (**B**) and sediment grain size (**C**). Letters above error bars indicate significant differences among meadows.

Seawater temperature was similar among meadows during the study period, but showed differences over time. Mean seawater temperatures increased from 25.27 ± 0.114 °C in July to 27.46 ± 0.120 °C in August but decreased to 16.33 ± 0.042 °C in October and to 14.12 ± 0.048 °C in November. Seawater salinity was different among meadows but did not show differences over time (Table 1). Overall, Meadow C showed lower salinity values than Meadows A and B along the study period (Figure 2A). 

The redox potential was similar among meadows along the study period (Table 1), ranging from −328.5 ± 28.18 to −244.6 ± 27.77 mV. The organic matter content was significantly different among meadows but did not change over time (Table 1), with Meadow A presenting higher values than Meadows B and C throughout the study period (Figure 2B). Regarding sediment grain size, the fine gravel content was similar among meadows (Table 1, Figure 2C), and the very coarse sand content was higher in Meadow A than in Meadow B but similar to that obtained in Meadow C (Table 1, Figure 2C). The coarse sand, medium sand, fine sand and very fine sand contents were very variable among meadows over time (Table 1, Figure 2C). However, the content of silt/clay was significantly higher in Meadow A than in other meadows during the study period (Table 1, Figure 2C). 

### 3.2. Seagrass Biometrics 

**Figure 3:**
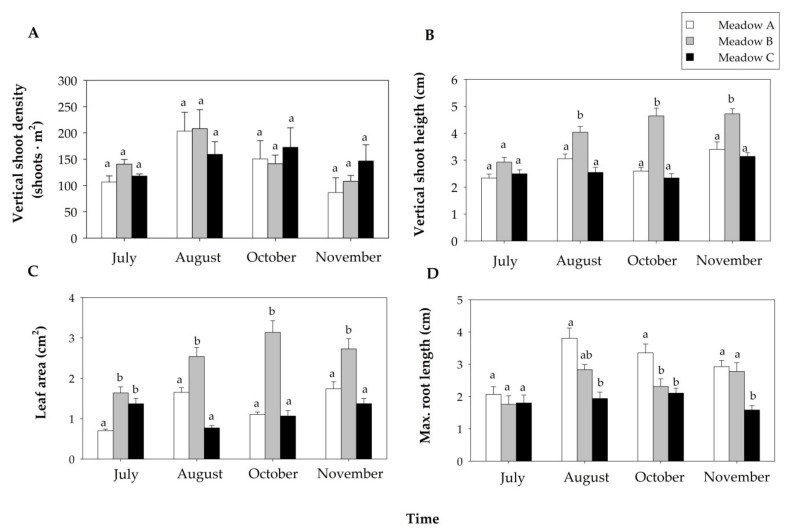
Seagrass biometrics (mean + SE) of the human-dominated *Z. noltei* meadows during the study period: vertical shoot density ((**A**), n = 3), vertical shoot height (n = 6, (**B**)), leaf area (n = 6, (**C**)) and maximum root length (n = 6, (**D**)) Different letters above error bars indicate significant differences among meadows over time.

The three *Z. noltei* meadows maintained similar vertical shoot density along the study period, but they showed significant differences over time (Table 1). Shoot density increased from July to August but decreased from August to November (Figure 3A).

The vertical shoot height, leaf area and maximum root length showed significant differences for the interaction between the factors meadow and time (Table 1). The vertical shoot height of the three meadows increased along the study period, with Meadow B showing the highest values (Figure 3B). The leaf area was especially variable in Meadows A and B over time, but Meadow B showed higher values than the other meadows during the study period (Figure 3C). In July, the three meadows exhibited similar values of maximum root length, but from August to November both Meadows A and B had longer roots than Meadow C (Figure 3D). 

### 3.3. Flowering Effort and the Relationship with Seagrass Biometrics and Abiotic Parameters

**Figure 4:**
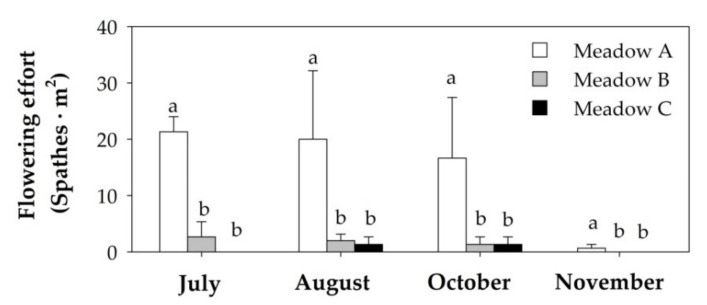
Flowering effort (mean + SE; n = 3) of the three studied human dominated *Z. noltei* meadows from July to November of 2019. Different letters above error bars indicate significant differences among meadows.

The flowering effort of the human-dominated *Z. noltei* meadows did not change over time but presented significant differences among meadows (Table 1). The flowering effort was significantly higher in Meadow A than in Meadows B and C throughout the study period (Figure 4). In addition, the production of sexual spathes persisted for a longer time in Meadow A than in other meadows (Figure 4).

**Figure 5:**
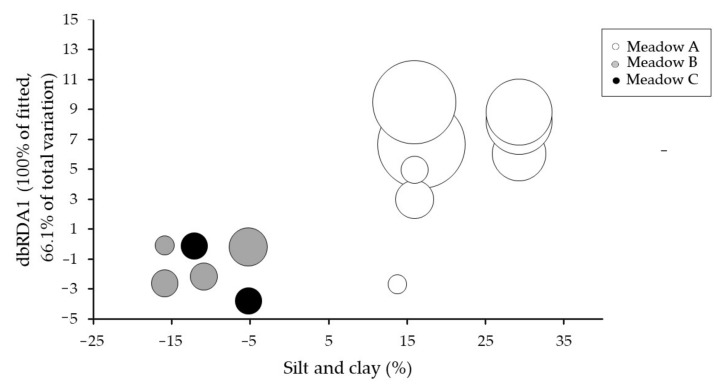
Distance-based redundancy (dbRDA) plot illustrating the DISTLM model based on the variability of the flowering effort over the study period considering the fitted seagrass biometrics and abiotic variables based on DISTLM analysis in Table 2. Bubble size represents the flowering effort of each seagrass meadow along the study period.

Sequential test of the DISTLM analyses showed that silt/clay and vertical shoot density were statistically significant (Table 2), with silt/clay explaining a greater amount of variation (40.77%) than the vertical shoot density (13.64%). Thus, these two variables were the best model to explain the variability in the flowering effort (Table 2, Figure 5). Since the organic matter content was strongly correlated with the silt/clay content, both variables could explain the same variability of the flowering effort.

## 4. Discussion

The production of sexual spathes in the human-dominated *Z. noltei* meadows of the Mira channel was strongly shaped by the vegetative shoot density of the seagrass and the silt and clay content of the sediment. Flowering effort was mostly induced in the closest area to the coastline (i.e., Meadow A) and was scarce near the oyster farm and upstream of the Mira channel (i.e., Meadows B and C). Moreover, these two areas showed lower organic matter and silt/clay content in the sediment than Meadow A. These results suggest that flowering in *Z. noltei* can vary depending on the sediment type and the organic matter content of the sediments.

Several studies have demonstrated the long-term adaptation of *Z. noltei* meadows inhabiting systems under anthropogenic pressure, including Ria de Aveiro [16,20,22]. Moreover, disturbances can stimulate the reproductive effort in certain seagrass species to guarantee their survival in the future [30]. Siltation and excessive loads of organic matter in the environment are two situations that can cause great stress in seagrasses [1]. Siltation can limit light availability and cause partial burial of the seagrass meadows affecting their development [52,53,54]. Organic matter enrichment is very common in areas where aquaculture is practiced and can promote anoxia in the sediment, stimulating anaerobic pathways that produce toxic substances for the seagrass meadows [55]. Thus, the greater siltation and organic matter load in Meadow A could have caused more pressure into this system, leading to an increased stress-induced flowering response. Similar results have been reported for *Zostera nigricaulis* (J. Kuo) in areas with high organic matter content and fine sediments [56]. In contrast, no spatial differences were found in the flowering effort of *Z. noltei* along a vertical gradient of different sediment grain-sizes in Ria Formosa, Portugal [57]. Nevertheless, the organic matter values in that coastal system were similar to those reported here in our study at Meadow A, which had the highest flowering effort, reinforcing our hypothesis that organic enrichment is a driver of stress-induced flowering in this seagrass.

*Zostera noltei* meadows are effective sinks of organic matter [45], since their below-ground structures can act as energy storage systems to support shoot production and flowering [58,59,60]. In fact, Meadow A presented the longest roots and the highest content of organic matter in the sediment, and evidenced the highest reproductive effort in our study. In contrast, Meadows B and C, established in sediments with low organic matter and silt and clay content, showed lower root length and production of sexual spathes during most of the reproductive period of the species. Organic matter mineralization is the major process for the supply of inorganic nitrogen and phosphorus to the porewater of marine sediments [1,61]; consequently, a higher content of organic matter could also lead to a higher concentration of inorganic nutrients in the sediment. This situation could be used as a strategy by the seagrass to store more nutrients in its below-ground structures and thus benefit its sexual reproduction [62]. In contrast, the low organic matter availability near the oyster farm and upstream of the channel could have limited stress-induced flowering responses in Meadows B and C. Therefore, content of fine sediments and accumulation of organic matter seem to be relevant for increasing resources dedicated to sexual reproduction in the genus *Zostera*. 

Under unfavorable conditions for flowering, clonal plants tend to predominate asexual versus sexual reproduction [62]. Clonal growth is a quicker and lower-risk tactic to maintain the survival and expansion of seagrasses than flowering [63,64]. However, the leaf and rhizome development of Meadows B and C, which barely developed sexual spathes in our study, was also differently affected over the flowering period. *Zostera noltei* inhabits areas of great spatial variability, and other environmental factors such as seawater temperature and salinity can influence its development [28,65]. The three studied meadows were intertidal and have been established in the Mira channel for over a decade [16]. Nevertheless, although they had been exposed to similar temperatures during the flowering period, they underwent different seawater salinity conditions. The tidal prism of the Mira channel results in more marine salinities at its mouth than in its upstream areas [66,67]. This effect was evidenced in the upstream *Z. noltei* meadow of our study, which showed lower salinities than the rest of the meadows. Although our analysis proved that this factor was not important for the flowering effort and vegetative shoot density of the seagrass, the leaves and the rhizomes of the upstream meadow barely grew during the reproductive period. In contrast, near the oyster farm, seawater salinity was euhaline (30–35 psu) and *Z. noltei* developed a larger leaf and rhizome growth than in the rest of the meadows. Thus, despite the *Z. noltei* ability to inhabit a wide range of salinities [24,25], the euhaline conditions near the oyster farm seemed to be more optimal for the human-dominated meadows than the lowest salinity ranges. However, since sexual reproduction only lasts a few months during the seagrasses’ annual cycle, a long-term study would be required to fully certify the status of these human-dominated meadows in the channel.

*Zostera noltei* is able to rapidly expand its coverage in human-dominated environments, but limitations on its reproductive effort could have serious long-term consequences. A greater flowering effort entails a greater stock of seeds in the area. In addition, the germination of these seeds is the only way that *Z. noltei* meadows have to recover against disturbances once the vegetative growth is slow or limited [68,69,70]. Then, monitoring the reproductive effort of seagrasses in areas where numerous anthropogenic activities simultaneously occur is essential for the conservation of these ecosystems. This knowledge can help us determine which seagrass areas are more susceptible to stress and require greater conservation management, but also to understand which areas may present a bottleneck for sexual reproduction and require a greater restoration effort with seeds or seedlings to increase their genetic variability.

In conclusion, the stress-induced flowering response of human-dominated *Z. noltei* meadows may depend on the environmental conditions of the area, but also on the vegetative development of the seagrass. The flowering effort of *Z. noltei* in human-dominated environments was triggered in silty sediments with high organic matter content, regardless of the anthropogenic pressure that acted in the area. Moreover, a greater investment in vegetative growth seems to limit the production of sexual spathes in this seagrass. However, more research on this topic is necessary to fully understand differences in the vegetative growth and flowering effort of *Z. noltei* on the Mira channel. Since increased flowering effort in seagrasses could indicate stressful situations, the information provided in this study is essential for the conservation of seagrass meadows inhabiting human-dominated environments.

## Figures and Tables

**Table 1:** Summary of the two-way ANOVA results [F (*p*-value)], including the factors *Meadow* and *Time*, for the flowering effort, seagrass biometrics and abiotic variables. Significant differences are highlighted in bold. Asterisks over variable names indicate that data did not fit normality and/or homogeneity of the variances and significant differences among treatments were adjusted to *p* < 0.01.

Variable	Meadow	Time	Meadow × Time
***Abiotic parameters***			
Temperature *	0.784 (0.479)	**4455.1 (<0.001)**	0.468 (0.820)
Salinity *	**21.87 (<0.001)**	2.094 (0.154)	0.337 (0.904)
Redox Potential	0.035 (0.966)	0.931 (0.455)	0.378 (0.879)
Organic matter	**39.07 (<0.001)**	1.165 (0.364)	1.339 (0.313)
Fine gravel	2.565 (0.118)	2.044 (0.161)	1.831 (0.175)
Very coarse sand *	**6.873 (0.010)**	1.689 (0.222)	0.288 (0.931)
Coarse sand	3.373 (0.069)	2.731 (0.122)	**3.428 (0.033)**
Medium sand	**116.0 (<0.001)**	0.577 (0.641)	**12.33 (<0.001)**
Fine sand	**14.71 (<0.001)**	0.877 (0.480)	**19.26 (<0.001)**
Very fine sand	**146.0 (<0.001)**	0.725 (0.556)	**10.17 (<0.001)**
Silt/Clay	**30.77 (<0.001)**	0.502 (0.688)	1.499 (0.259)
***Seagrass biometrics***			
Vertical shoot density	0.309 (0.737)	**5.413 (0.005)**	0.985 (0.457)
Vertical shoot height ^a^	**60.54 (<0.001)**	**17.83 (<0.001)**	**3.770 (0.001)**
Leaf area ^b^	**90.00 (<0.001)**	**12.62 (<0.001)**	**12.53 (<0.001)**
Maximum root length ^a^	**26.25 (<0.001)**	**12.37 (<0.001)**	**2.918 (0.009)**
Flowering effort *	**10.41 (0.001)**	1.693 (0.195)	1.112 (0.384)

^a^ Ln (x + 1) transformed. ^b^ Log (x) transformed.

**Table 2:** Results obtained in the sequential test of the DISTLM analysis between the flowering effort, seagrass biometrics and abiotic parameters. Proportion: proportion of the variation in flowering effort explained by each variable; Cum. %: cumulative percentage of variance explained. Significant variables are highlighted in bold.

Variable	Variance %	*F*	*p*	Cum. %
Silt/Clay/Organic matter	40.77	23.40	**0.001**	40.77
Vertical shoot density	13.64	9.873	**0.007**	54.41
Very coarse sand	3.535	2.689	0.091	57.94
Vertical shoot height	0.634	0.474	0.488	58.58
Leaf area	1.933	1.469	0.230	60.51
Temperature	1.410	1.074	0.301	61.92
Maximum root length	1.113	0.843	0.359	63.03
Salinity	0.847	0.633	0.433	63.88
Very fine sand	1.116	0.829	0.378	65.00
Medium sand	0.635	0.462	0.517	65.63
Redox potential	0.258	0.182	0.672	65.89
Coarse sand	0.061	0.041	0.855	65.95
Fine sand	0.123	0.080	0.778	66.07
Fine gravel	0.000	0.000	1.000	66.07
